# Coculture with hemicellulose-fermenting microbes reverses inhibition of corn fiber solubilization by *Clostridium thermocellum* at elevated solids loadings

**DOI:** 10.1186/s13068-020-01867-w

**Published:** 2021-01-18

**Authors:** Dhananjay Beri, Christopher D. Herring, Sofie Blahova, Suresh Poudel, Richard J. Giannone, Robert L. Hettich, Lee R. Lynd

**Affiliations:** 1 Thayer School of Engineering, Dartmouth College, Hanover, NH 03755 USA; 2 Centre for Bioenergy Innovation, Oak Ridge National Laboratory, Oak Ridge, TN 37830 USA; 3 Enchi Corporation, Lebanon, NH 03766 USA; 4 Chemical Sciences Division, Oak Ridge National Laboratory, Oak Ridge, TN 37830 USA

## Abstract

**Background:**

The cellulolytic thermophile *Clostridium thermocellum* is an important biocatalyst due to its ability to solubilize lignocellulosic feedstocks without the need for pretreatment or exogenous enzyme addition. At low concentrations of substrate, *C. thermocellum* can solubilize corn fiber > 95% in 5 days, but solubilization declines markedly at substrate concentrations higher than 20 g/L. This differs for model cellulose like Avicel, on which the maximum solubilization rate increases in proportion to substrate concentration. The goal of this study was to examine fermentation at increasing corn fiber concentrations and investigate possible reasons for declining performance.

**Results:**

The rate of growth of *C. thermocellum* on corn fiber, inferred from CipA scaffoldin levels measured by LC–MS/MS, showed very little increase with increasing solids loading. To test for inhibition, we evaluated the effects of spent broth on growth and cellulase activity. The liquids remaining after corn fiber fermentation were found to be strongly inhibitory to growth on cellobiose, a substrate that does not require cellulose hydrolysis. Additionally, the hydrolytic activity of *C. thermocellum* cellulase was also reduced to less-than half by adding spent broth. Noting that > 15 g/L hemicellulose oligosaccharides accumulated in the spent broth of a 40 g/L corn fiber fermentation, we tested the effect of various model carbohydrates on growth on cellobiose and Avicel. Some compounds like xylooligosaccharides caused a decline in cellulolytic activity and a reduction in the maximum solubilization rate on Avicel. However, there were no relevant model compounds that could replicate the strong inhibition by spent broth on *C. thermocellum* growth on cellobiose. Cocultures of *C. thermocellum* with hemicellulose-consuming partners—*Herbinix* spp. strain LL1355 and *Thermoanaerobacterium thermosaccharolyticum*—exhibited lower levels of unfermented hemicellulose hydrolysis products, a doubling of the maximum solubilization rate, and final solubilization increased from 67 to 93%.

**Conclusions:**

This study documents inhibition of *C. thermocellum* with increasing corn fiber concentration and demonstrates inhibition of cellulase activity by xylooligosaccharides, but further work is needed to understand why growth on cellobiose was inhibited by corn fiber fermentation broth. Our results support the importance of hemicellulose-utilizing coculture partners to augment *C. thermocellum* in the fermentation of lignocellulosic feedstocks at high solids loading.

## Introduction

Corn fiber represents a “Generation 1.5” biofuel feedstock, intermediate between starch and lignocellulose. Since it is already present at corn-based biofuel facilities, a corn fiber-based process can potentially be added to an existing corn ethanol plant in a ‘bolt-on’ configuration [1]. It therefore represents an important possible opportunity to demonstrate thermophiles as a novel, low-cost cellulosic fuel technology.

Consolidated bioprocessing (CBP) using the cellulolytic thermophile *Clostridium thermocellum* can potentially reduce the cost of cellulosic ethanol production by eliminating the need for exogenous enzymes [1–3]. Recent innovations suggest that CBP using thermophilic organisms can be combined with milling during fermentation (cotreatment) and can achieve highly efficient deconstruction of biomass without the need for expensive pretreatment [4]. *C. thermocellum* deconstructs lignocellulosic plant biomass with a multi-enzyme cellulosome expressed on its cell surface [2, 5]. In addition to cellulases for breaking down cellulose into cellodextrins, which *C. thermocellum* utilizes for its growth, the cellulosome also comprises enzymes to breakdown hemicellulose [6, 7]. However, *C. thermocellum* is unable to utilize hemicellulose hydrolysis products [8].

To minimize the cost of distillation, it is important that ethanol fermentations reach an ethanol concentration of 40 g/L [9, 10]. This requires carbohydrate concentrations of at least 80 g/L, or lignocellulose concentrations of at least 120 g/L. Allowing for less-than theoretical solubilization and fermentation, a lignocellulose concentration ≥ 150 g/L is a realistic expectation for an industrial process. Handling and mixing biomass at these concentrations are challenging in batch culture [10–12], although biomass slurries undergo dramatic liquefaction in the early stages of biologically mediated solubilization, favoring fed-batch or continuous configurations. Cultivation of *C. thermocellum* at biomass loadings anticipated for an industrial process has not been reported to our knowledge, although high solubilization of pure cellulose at loadings up to 120 g/L has been documented [13]. Basen et al. looked at the fermentation performance of another potential CBP organism, *Caldicellulosiruptor bescii*, on untreated switchgrass at industrially relevant biomass loadings. They observed 30% solubilization at 50 g/L solids loading and substantial growth and solubilization even at 200 g/L. They suggest that further fermentation of switchgrass was inhibited by organic acid end-products and by a specific inhibitor produced during the fermentation of untreated switchgrass by *C. bescii* that did not affect other thermophilic bacteria [14].

Many studies have examined enzymatic hydrolysis of plant biomass at high solids loading and it is generally accepted that the product yield decreases at high solids loading, a phenomenon referred to as the ‘solids effect’ [15]. For cell-free fungal cellulase preparations, which have been most widely studied in this context, this effect generally becomes pronounced at solids loadings ≥ 150 g/L [12, 15]. Proposed mechanisms driving this effect include inhibitors released during pretreatment or hydrolysis, as well as physical limitations such as water availability and enzyme adsorption characteristics [12, 15]. Possible biomass-derived inhibitors can include phenolic compounds, furan derivatives or weak acids, such as acetate [14, 16, 17]. Soluble carbohydrates from hydrolysis of hemicellulose, such as short chain oligomers of xylans or mannans, have also been found to inhibit cellulase activity in multiple studies [18–21].

A trend of declining solubilization with increasing solids has been reported at substantially lower solids loading for fermentation of lignocellulose by *C. thermocellum* cultures [10, 22, 23]. Verbeke et al. looked at the solubilization yield on switchgrass at 10, 25, or 50 g/L solids loading, and compared it to a model crystalline cellulose (Avicel). Total cellulose solubilization for switchgrass was measured as 63%, 47%, and 37% with increasing solids, while the Avicel was solubilized completely in all cases. The spent broth from 50 g/L switchgrass fermentation was found to inhibit *C. thermocellum* growth on Avicel but not on cellobiose. It was suggested that the likely reason for inhibition is xylooligosaccharides (XOS) liberated from the switchgrass that accumulate in the liquid broth [23]. XOS are strong inhibitors of fungal cellulases [21], but there are no studies showing their effect on cellulases from *C. thermocellum*. Other potential growth inhibitors such as ferulic acid can also be liberated from biomass [24], while fermentation products such as ethanol or lactate are also known to be inhibitory [16].

In this study, we investigated the performance of *C. thermocellum* with increasing solids loading of corn fiber, with a focus on understanding the role of hemicellulose. Corn fiber, the fibrous portion of corn kernels, has almost no lignin and a high hemicellulose-to-cellulose ratio [25], and is expected to release a substantial amount of hemicellulose oligomers in fermentation, even at moderate solids loading. We recently reported structural characterization of corn fiber hemicellulose released by *C. thermocellum* [26]. While the glucuronoxylan carbohydrate was recalcitrant to most bacteria we tested, we identified a novel isolate *Herbinix* spp. strain LL1355 that can consume more than 85%, enabling almost complete solubilization of corn fiber by *C. thermocellum* at low solids loading.

## Results and discussion

### *Clostridium thermocellum* bioreactor fermentations on corn fiber

**Fig. 1:**
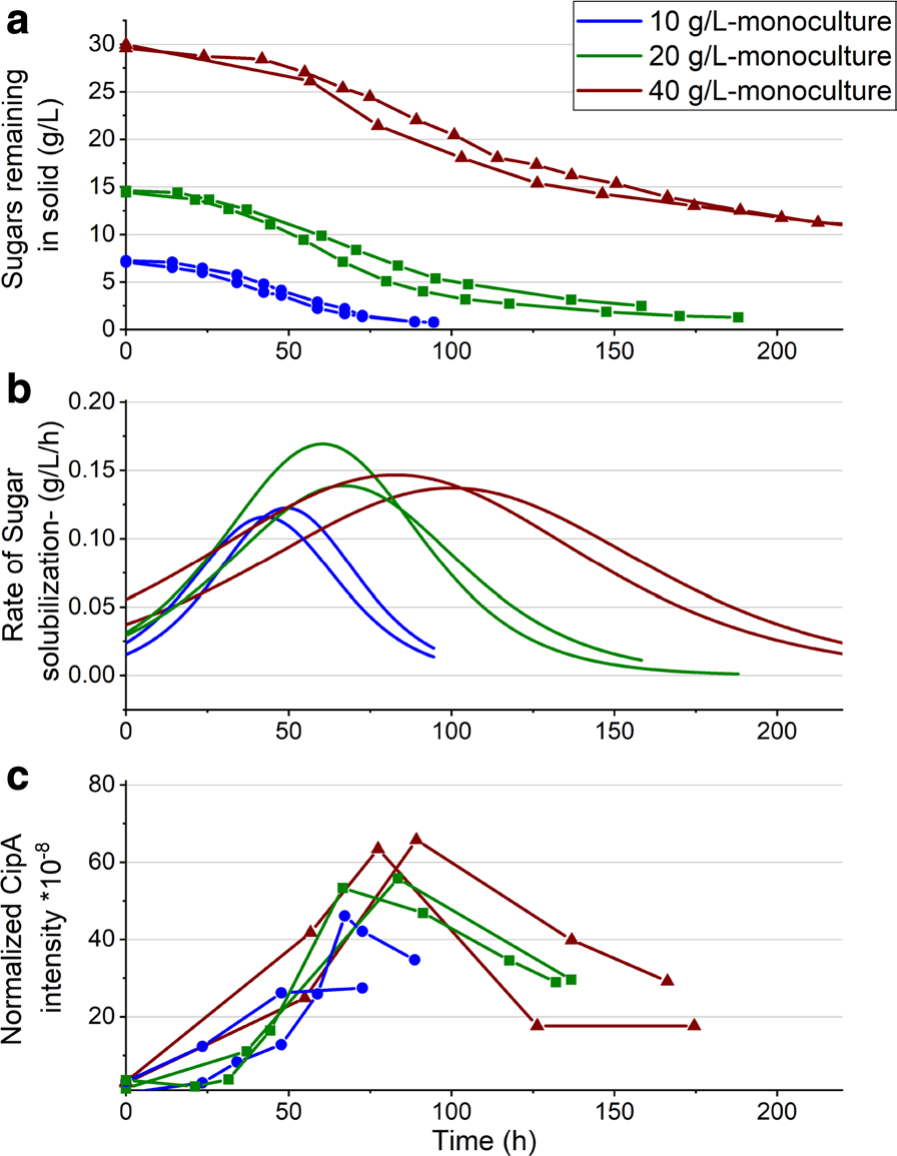
*C. thermocellum* fermentations on corn fiber. Fermentation data for *C. thermocellum* monoculture fermentations with increasing solids loading of corn fiber: **a** sugar-equivalent carbohydrate remaining in the solid fraction; **b** rate of solubilization; **c** normalized CipA protein amount with increasing solids loading

Previous studies have documented the performance of *C. thermocellum* in fermentations at various solids loading in small-scale bottle experiments [22, 23]. To expand and improve upon them, we ran corn fiber fermentations in bioreactors to allow for pH control, regular sampling, and measurement of the rate of solubilization. The data from duplicate fermentations at 10, 20, and 40 g/L are shown in Fig. 1. Compositional analysis of the corn fiber used in these fermentations showed carbohydrate equivalent to 77% sugars, including 13.8% arabinose, 39.1% xylose + galactose, and 24.3% glucose.

At a solids loading of 10 g/L, *C. thermocellum* solubilized 90% of the sugars in 90 h, eventually reaching 95% solubilization in 5 days. This is greater than the extent of solubilization of switchgrass at low solids loading in the work of Balch et al. [4], even without cotreatment. The cultures at 20 g/L reached 90% solubilization after 180 h. However, the 40 g/L fermentation only reached 67% solubilization even after 280 h, after which no further solubilization occurred. A Boltzmann function sigmoidal curve was fit to the data in Fig. 1a, with *R*^2^ values greater than 0.99 in all cases. These curves were differentiated to determine the rate of solubilization, as shown in Fig. 1b. The maximum rate of solubilization remained roughly the same with increasing solids loading. This is in contrast to the performance of *C. thermocellum* on the purified cellulose Avicel shown by Holwerda et al. [27], wherein the maximum rate showed an approximately linear increase between 5 and 70 g/L Avicel loading. With all loadings of corn fiber, the maximum solubilization rate was near 0.13 g/L/h, and occured when about half of the final solubilization had taken place.

Typical methods to measure biocatalyst levels such as optical density or nitrogen analysis could not be used in these fermentations due to interference from the feedstock, so we performed LC–MS/MS proteomics analysis on centrifuged samples, using the cellulosome scaffoldin protein CipA in washed sample pellets as an indicator of active biocatalyst (Fig. 1c) [28]. There was negligible increase in cell amount with increasing solids loading. The maximum cell density coincided with the maximum solubilization rate for all fermentations. At 40 g/L, it is striking that after 90–100 h, the cell population ceased to grow and the solubilization rate declined when more than 2/3 of the initial carbohydrate still remained in the solids.

### Effect of spent broth on *C. thermocellum* and its cellulase

To investigate these phenomena, we first tested the effect of spent broth, collected at various times during the 40 g/L corn fiber fermentation, on *C. thermocellum* growth on cellobiose. A control broth was prepared from a *C. thermocellum* fermentation on 4.2 g/L Avicel, which was approximately equal to the total amount of glucan consumed during the 40 g/L corn fiber fermentation. This broth controlled for fermentation products such as ethanol that are normally present in the spent broth. The *C. thermocellum* strain used in this experiment was LL1043, from which lactate dehydrogenase, phosphotransacetylase, and acetate kinase have been deleted. While the strain itself produces negligible amounts of acetate or lactate, some acetate was released from corn fiber.

**Fig. 2:**
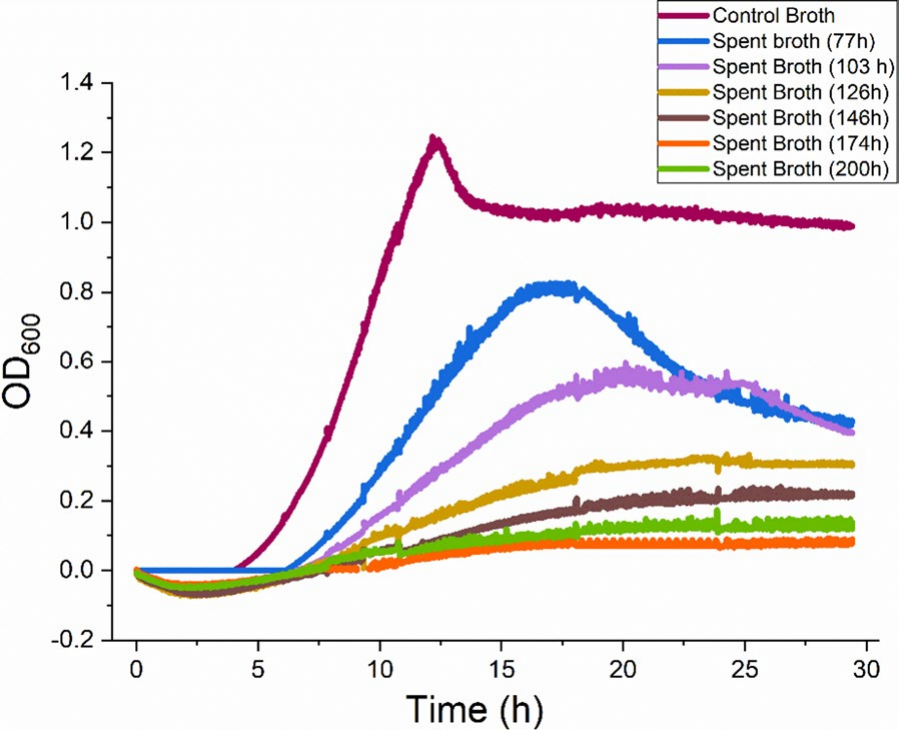
Inhibition of *C. thermocellum* growth on cellobiose. Growth of *C. thermocellum* on cellobiose with the addition of spent broths sampled at various times from a 40 g/L monoculture fermentation on corn fiber. Cultures and measurements of optical density at 600 nm (OD_600_) were conducted in a plate reader. Spent broth constituted 75% of the cultures by volume, with the remainder being cellobiose, media components, and inoculum

**Fig. 3:**
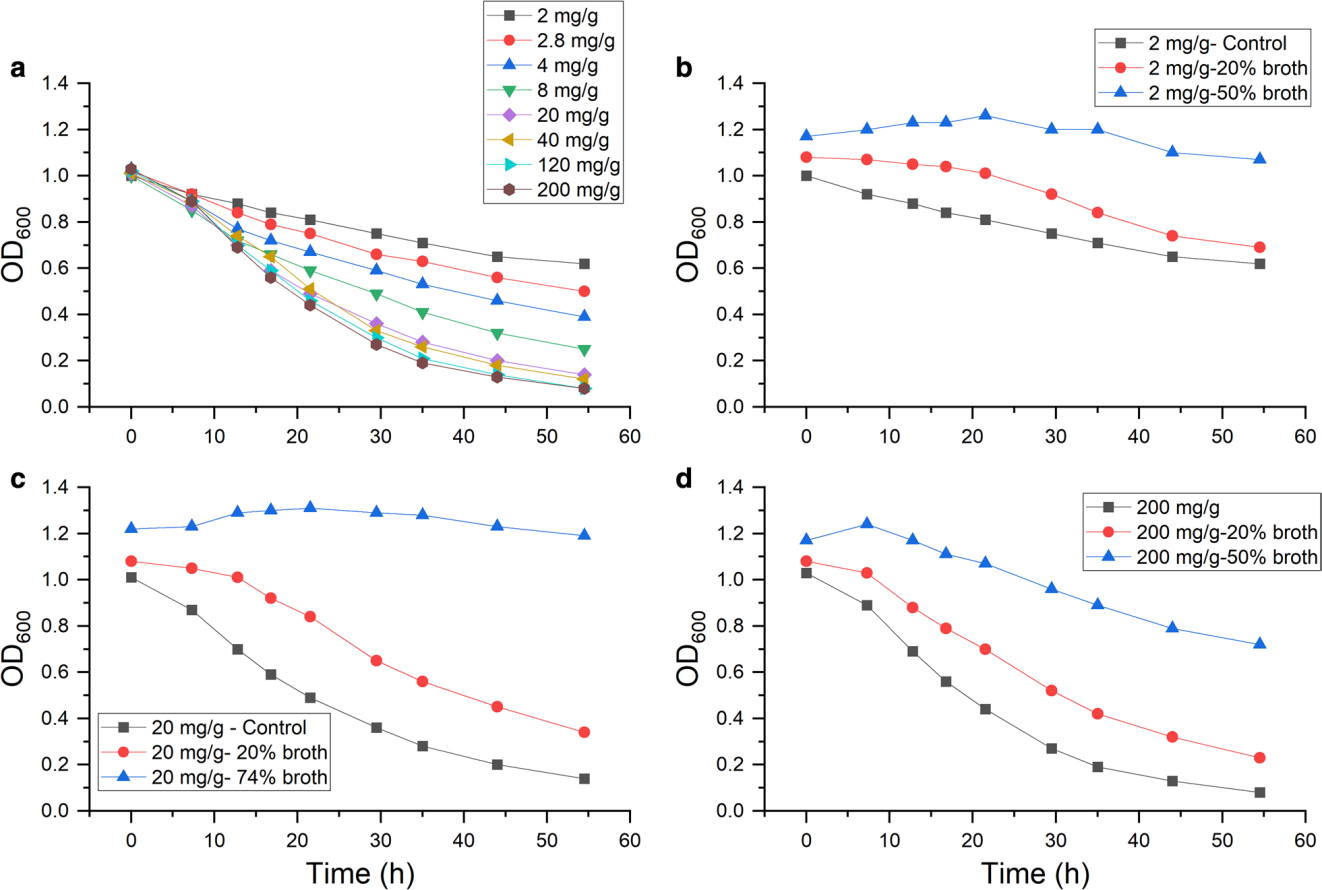
Inhibition of *C. thermocellum* cellulase. Cellulase enzyme activity assay with starting concentration of 1 g/L Avicel and reaction volume of 5 mL. Legend shows amount of enzyme in mg (cellulase)/g (Avicel) and the amount of spent broth added. The cellulase enzyme was prepared by concentrating the supernatant from a *C. thermocellum* fermentation on 20 g/L Avicel. **a** Dependence of activity on amount of added enzyme; **b**–**d** inhibition of cellulase activity with different amounts of added enzyme and broth

Figure 2 shows the optical density of these cellobiose cultures over time. Spent broth taken from corn fiber fermentation became increasingly inhibitory as the fermentation progressed. The broths from 126 and 146 h of corn fiber fermentation were highly inhibitory, while the broths from 174 and 200 h completely inhibited growth on cellobiose. The inhibition suggests a toxicity that may explain the drop in biocatalyst levels after 90–100 h in the 40 g/L corn fiber reactor in Fig. 1c. This result is in contrast to a previous study by Verbeke et al. [21] that had shown no inhibition of *C. thermocellum* growth on cellobiose by spent broths from a 50 g/L switchgrass fermentation. The difference may be due to the much higher levels of hemicellulose in corn fiber relative to switchgrass or the presence of other inhibitors in corn fiber that are absent in switchgrass. At higher solids loading (e.g., 100 g/L), the broth from switchgrass fermentations may also become inhibitory to *C. thermocellum* growth on cellobiose, although this remains to be shown. To study the effect of the spent broth on *C. thermocellum*’s hydrolytic activity, we analyzed the effect of spent broth on cellulase hydrolysis in vitro using cellulase prepared by high-molecular-weight cut-off filtration. The spent broth used for these experiments was collected after 250 h of corn fiber fermentation. The cellulase enzyme activity assay, adapted from Johnson et al. [29], was based on the monitoring of residual Avicel concentration by measuring the optical density at 600 nm. Figure 3 shows the disappearance of Avicel versus time with different amounts of *C. thermocellum* cellulase added. A titration of added cellulase shown in Fig. 3a shows that hydrolysis was maximized with 20 mg/g cellulase. Figure 3b shows that for 2 mg/g of enzyme, 50% broth addition almost completely inhibited Avicelase activity. Figure 3c shows that for 20 mg/g of enzyme, 74% broth addition had the same effect. Even at ten times the saturation enzyme level, with 200 mg/g of enzyme, 50% broth substantially reduced the enzyme activity (Fig. 3d).

For a quantitative measurement of the rate, the glucose release was measured at multiple sampling times (Additional file 1: Figure S1). After 5.5 h of incubation, with 50% addition of broth, the glucose release was found to be 45 ± 5% (*n* = 2) of the control.

### Inhibitory effects of various carbohydrates

The data presented above demonstrate that one or more components of corn fiber spent broth were inhibitory to *C. thermocellum* growth and its cellulase. Given that XOS have been shown to be inhibitory to fungal cellulases [21], a likely candidate is arabinoxylan oligosaccharides, indicated by concentrations up to 15 g/L in a fermentation of 40 g/L corn fiber (Additional file 1: Figure S2). We investigated this possibility by testing the effects of various carbohydrates on cellular growth via cellobiose cultures, on cellulase activity via enzyme assays, or on the combination of both, via Avicel cultures.

#### Effect on *C. thermocellum* growth on cellobiose

First, a range of commercially available carbohydrates were tested for their effect on *C. thermocellum* growth in a plate reader on cellobiose in concentration of up to 10 g/L or 20 g/L. These included: (i) XOS (degree of polymerization of 2–6) in a mixture as well as separately; (ii) wheat arabinoxylan and arabinoxylan oligosaccharides; (iii) starch hydrolysis products such as maltose, maltodextrin, and pullulan; (iv) xyloglucan and xyloglucan oligosaccharides. All monosaccharides present in corn fiber were also tested. The full list of 28 tested compounds is shown in Additional file 1: Table S1.

No growth inhibition was observed for the compounds tested, except xylose, mannose, and ribose. Verbeke et al. [30] tested monosaccharides and oligomers for their effect on the rate of *C. thermocellum* growth on cellobiose. They showed that xylobiose caused a > 50% reduction in growth rate at 20 g/L, while other XOS were slightly less inhibitory. They also showed that xylose affected growth rate at a similar level, with 50% reduction at 20 g/L. While we also observed inhibition with xylose (57 ± 2% and 33 ± 3% reduction at 20 and 10 g/L, respectively, *n* = 4), we did not observe inhibition by XOS at up to 20 g/L. As d-ribose was reported to be a strong inhibitor by Verbeke et al., we tested that at 20 g/L and found a 32 ± 2% reduction in growth rate. At a 10 g/L concentration of d-ribose, there was no effect. Mannose was also an interesting case, as it caused a lag phase for 2–3 days, after which the growth rate was normal.

Importantly, our experiments showed no growth effects from arabinoxylan oligosaccharides and XOS, which are the commercially available compounds most closely related to the corn arabinoxylan oligomers found in spent broth. Xylose was found to be inhibitory, but since the broth only has small amounts of monomeric xylose (~ 1 g/L), it is unlikely to be the primary cause of inhibition.

#### Inhibition of *C. thermocellum* cellulase activity

**Fig. 4:**
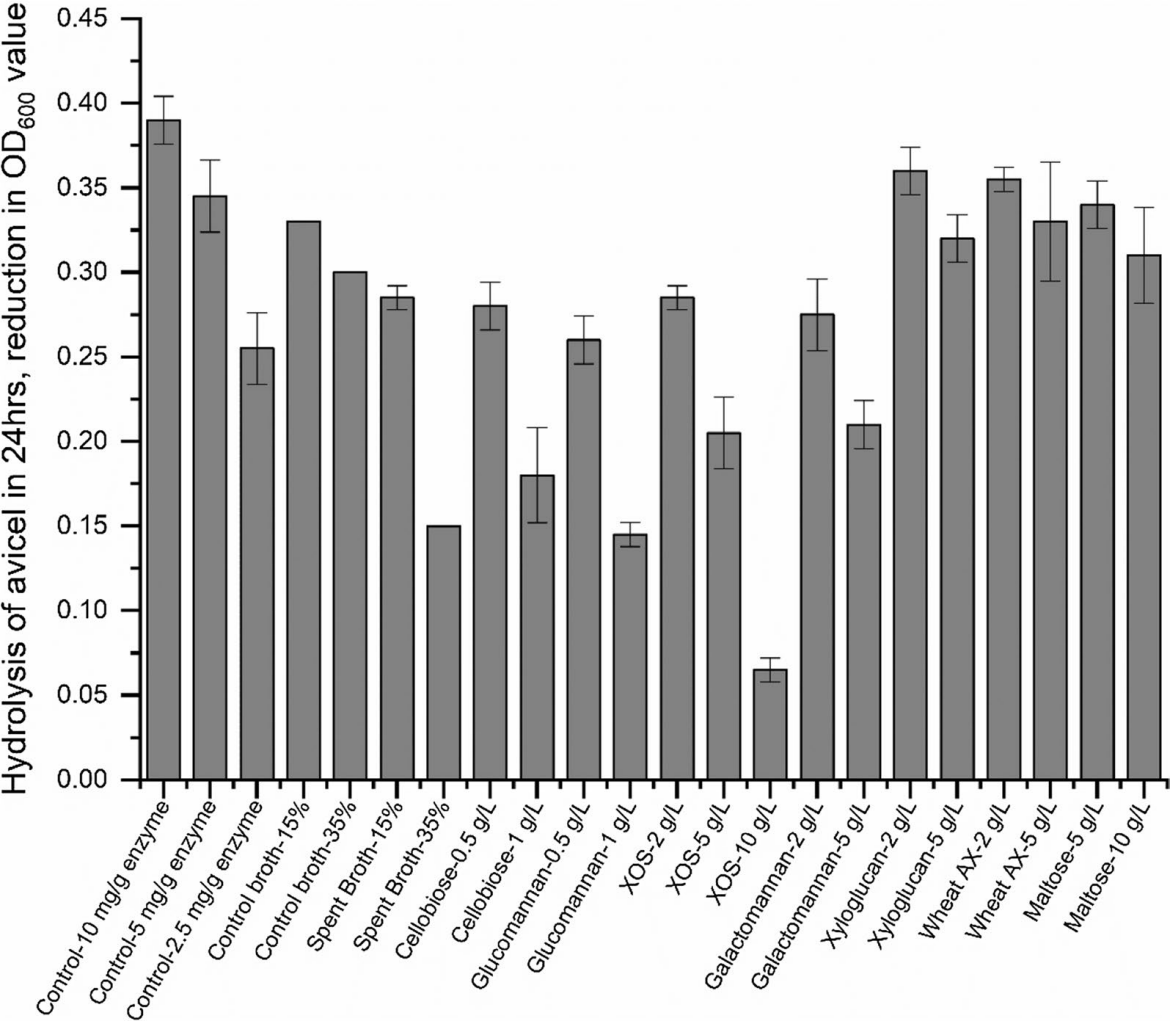
Effect of different carbohydrates on *C. thermocellum* cellulase activity. 0.5 g/L initial Avicel was incubated with 10 mg/g *C. thermocellum* cellulase and activity was estimated by measuring the decrease in OD_600_ after 24 h. The cellulase enzyme was prepared by concentrating the supernatant from a *C. thermocellum* fermentation on 20 g/L Avicel. The error bars show the SD for *n* = 2. *XOS* xylooligosaccharides, *AX* arabinoxylan, control broth—see “Materials and methods”. Glucomannan, galactomannan, and xyloglucan used here are all oligosaccharide mixtures, prepared by partial enzymatic hydrolysis of the respective carbohydrate

We next performed qualitative enzyme activity assays to test the effect of different carbohydrates on *C. thermocellum* cellulase, by measuring the OD_600_ over time, as described above. Figure 4 shows the decrease in OD_600_ value after 24 h. To broaden the scope of this study, we decided to look at a large variety of hemicelluloses that are commonly found in lignocellulosic feedstocks for biofuel production including woods and grasses. The effect of these carbohydrates on *C. thermocellum* cellulases has not been studied previously. The effect of reduced enzyme loading was also tested. Cellobiose is a known inhibitor of cellulases [31] and we found it to be a strong inhibitor of *C. thermocellum* cellulase in our study, as well. Fortunately, *C. thermocellum* rapidly consumes any cellobiose produced by its enzymes and we have not seen it accumulate in the broth of an actively growing culture. As shown in Fig. 4, cellobiose reduced the rate of hydrolysis to less-than half with 1 g/L addition. Looking at carbohydrates that are similar to those found in the spent broth, XOS were a moderately strong inhibitor of the *C. thermocellum* cellulases as well, similar to what has been reported previously for fungal cellulases [18, 21, 32]. XOS were not as inhibitory as cellobiose, needing 5 g/L addition to halve the activity. 35% addition of spent broth, which was equivalent to adding 5 g/L of carbohydrates (as measured by HPLC of acid hydrolyzed broth, i.e., liquid QS), reduced activity to an extent similar to that of 5 g/L XOS. This suggests that the inhibition of *C. thermocellum* cellulases by the spent broth may be explained by the presence of XOS-like structures. Importantly, arabinoxylan (AX), which has not been evaluated in previous studies of cellulase inhibition to the best of our knowledge, caused less inhibition than XOS. This could be due to the higher degree of polymerization (DP) of the Wheat AX used. Beechwood and Birchwood xylan, which also had high DP, showed inhibition similar to Wheat AX (data not shown). Similarly, xyloglucan oligosaccharides, which are present in corn fiber and spent broth from *C. thermocellum* fermentation, caused only moderate inhibition. Galactomannan also showed a moderate inhibitory effect. No monosaccharide caused inhibition (Additional file 1: Figure S3).

Surprisingly, we found glucomannan to be a potent inhibitor, as strong as cellobiose. Kumar et al. [19] have previously shown strong inhibition of fungal cellulases by mannan polysaccharides, but mannans form viscous solutions and it is difficult to ascertain the role of viscosity in the reduction of cellulase activity that is reported. We prepared glucomannan oligosaccharides by hydrolyzing the polysaccharide with β-mannanase and then removing the large polymers with ethanol precipitation, thus obtaining a non-viscous oligosaccharide solution, which we found to be inhibitory. The inhibition of *C. thermocellum* cellulase activity by galactomannan oligosaccharides was much less, in contrast to the fungal cellulase results from Kumar et al. who reported more inhibition with galactomannan than glucomannan.

This experiment was also performed using a quantitative enzyme assay, as for the Spent Broth (see “Materials and methods”), based on measuring the glucose released by the enzyme from Avicel. However, the amount of glucose released was up to an order of magnitude lower than the glucose present in the added carbohydrate, so error in measurement was high. Data are not shown here, though the trends were the same as presented in Fig. 4.

#### Effect on *C. thermocellum* growth on Avicel

**Fig. 5:**
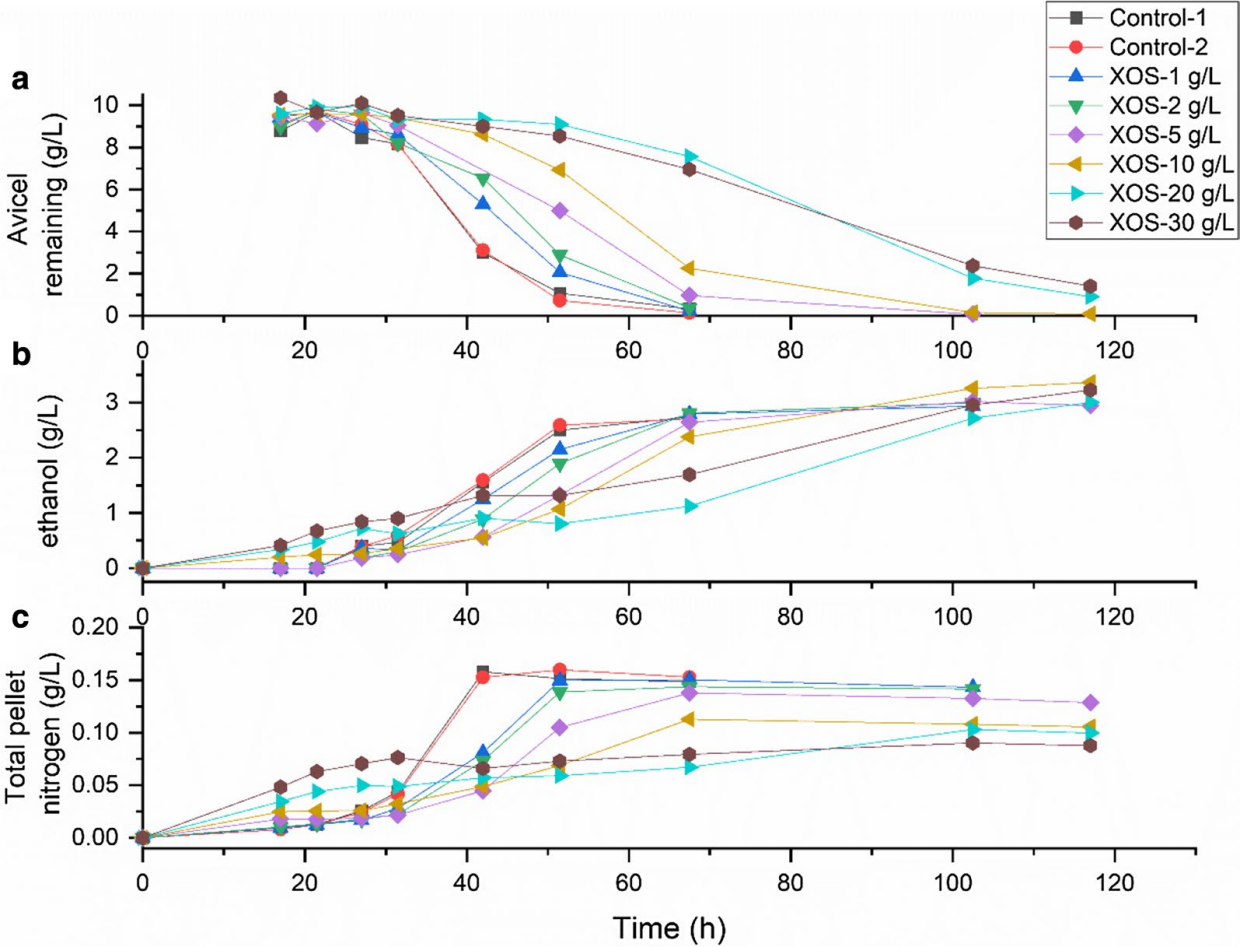
Effect of XOS addition on growth of *C. thermocellum* on Avicel. Fermentations were done in serum bottles with starting concentration of 10 g/L Avicel. **a** Avicel remaining (g/L), **b** ethanol produced (g/L), and **c** cell growth estimated by total pellet nitrogen (g/L)

The effect of commercially available hemicellulose carbohydrates was then tested by growing *C. thermocellum* on Avicel with the addition of various carbohydrates. Growth rate was monitored both by measuring ethanol production as well as residual Avicel during the time-course of the culture with increasing amounts of XOS (Fig. 5). Growth inhibition is evident, especially at 20 and 30 g/L XOS. At these concentrations, Avicel was not completely utilized after 5 days, whereas it was completely consumed after 3 days for the controls. Ethanol production was also slowed down with increasing XOS.

Final ethanol concentrations were slightly higher for cultures containing XOS, possibly due to the presence of a small amount of sugar fermentable by *C. thermocellum* in the XOS. This would also explain why ethanol production and cell growth starts sooner in these cultures without Avicel having been consumed. Figure 5c shows an initial spurt of cell growth in the cultures with 20 or 30 g/L XOS but then no subsequent growth, as would be expected if soluble sugar was immediately consumed, but cellulose hydrolysis was completely inhibited. A similar phenomenon may have occurred during growth on 40 g/L corn fiber fermentation (Fig. 1). To ascertain that the lack of pH control did not have a role to play in the observed XOS inhibition, we conducted bioreactor fermentations with 20 g/L Avicel, with and without the addition of 20 g/L XOS (Additional file 1: Figure S4). The data showed a dramatic reduction in growth rate with XOS addition and a halving of the maximum cell density.

**Fig. 6:**
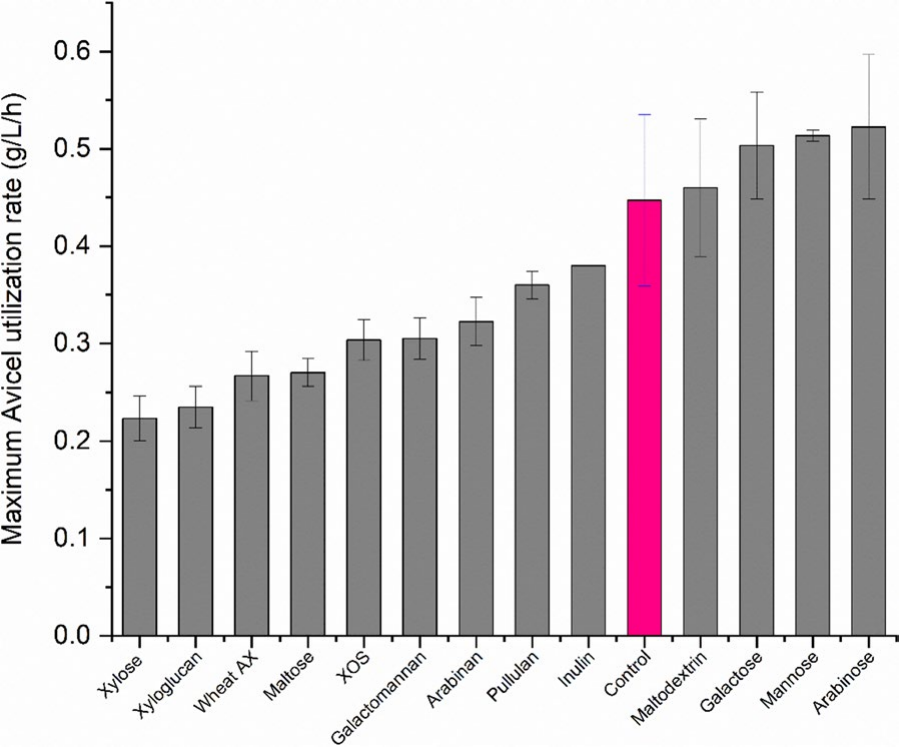
Effect of various carbohydrates on *C. thermocellum* growth on Avicel. *C. thermocellum* was grown on 10 g/L Avicel with the addition of 10 g/L of various carbohydrates. Rate was calculated by differentiating a Boltzmann Sigmoidal fit curve on the Avicel utilization time-course data. *R*^2^ values of > 0.98 were observed in all cases. Error bars show the standard deviation (*n* ≥ 2). For the control *n* = 6. *XOS* Xylooligosaccharides, *AX* arabinoxylan

Many other carbohydrates were tested for their effects on Avicel fermentation, with results summarized in Fig. 6. The maximum Avicel utilization rate was calculated in these tests by differentiating a Boltzmann Sigmoidal fit of the utilization curves (Additional file 1: Figure S5 and Additional file 2). The results were expected to closely parallel results from the cellulase assays, as the inhibition of growth on Avicel might be expected to be largely explained by the inhibition of cellulases. Xyloglucan, wheat AX, galactomannan, maltose, and XOS all reduced growth rate to a similar degree as observed for cellulase inhibition. Xyloglucan, maltose, and wheat AX had not shown much inhibition in the cellulase enzyme assays, but did significantly reduce growth rate. It is possible that the inhibition by wheat AX and xyloglucan was due to the hydrolysis of large molecules into lower DP oligomers by *C. thermocellum* enzymes during the long growth period. Higher concentration of these molecules would be expected to cause more inhibition. The cellulase assays measured the enzyme activity after 6 h which may not have been enough to hydrolyze the xyloglucan and wheat AX. For maltose, it is conceivable that this sugar interfered with cellodextrin transport, which would explain why inhibition was observed for growth on Avicel but not for cellulase activity or growth on cellobiose.

The monosaccharides mannose, galactose, and arabinose actually led to a small increase in growth rate. As with growth on cellobiose, mannose caused a long lag phase on Avicel too, which gradually decreased after transferring the strain a few times in medium-containing mannose. The inhibitory effect of xylose on *C. thermocellum* growth on Avicel mirrors its inhibition of growth rate on cellobiose (described above). Glucomannan, which was found to be a strong inhibitor of cellulase but had no effect on growth on cellobiose, completely inhibited growth of *C. thermocellum* on Avicel when present at 2 g/L, and substantially reduced the growth rate even at 0.2 g/L. This may be an important observation for fermentations of woody feedstocks like poplar and pine that are known to contain a substantial amount of glucomannan [33].

### Performance of cocultures of *C. thermocellum* with hemicellulose-fermenting microbes

Our results indicate that soluble hemicellulose hydrolysis products inhibit cellulase activity and Avicel growth more than growth on cellobiose. To test if consuming the hemicellulose that is liberated from corn fiber by *C. thermocellum* can alleviate inhibition*,* we ran ternary cocultures with hemicellulose-utilizing thermophiles *Herbinix* spp. strain LL1355 and *T. thermosaccharolyticum* ethanologen strain LL1548*.* Although LL1355 can consume most of the hemicellulose without *T. thermosaccharolyticum*, we included *T. thermosaccharolyticum* to reduce formation of lactic acid, the main product of fermentation by *Herbinix* spp. strain LL1355.

**Fig. 7:**
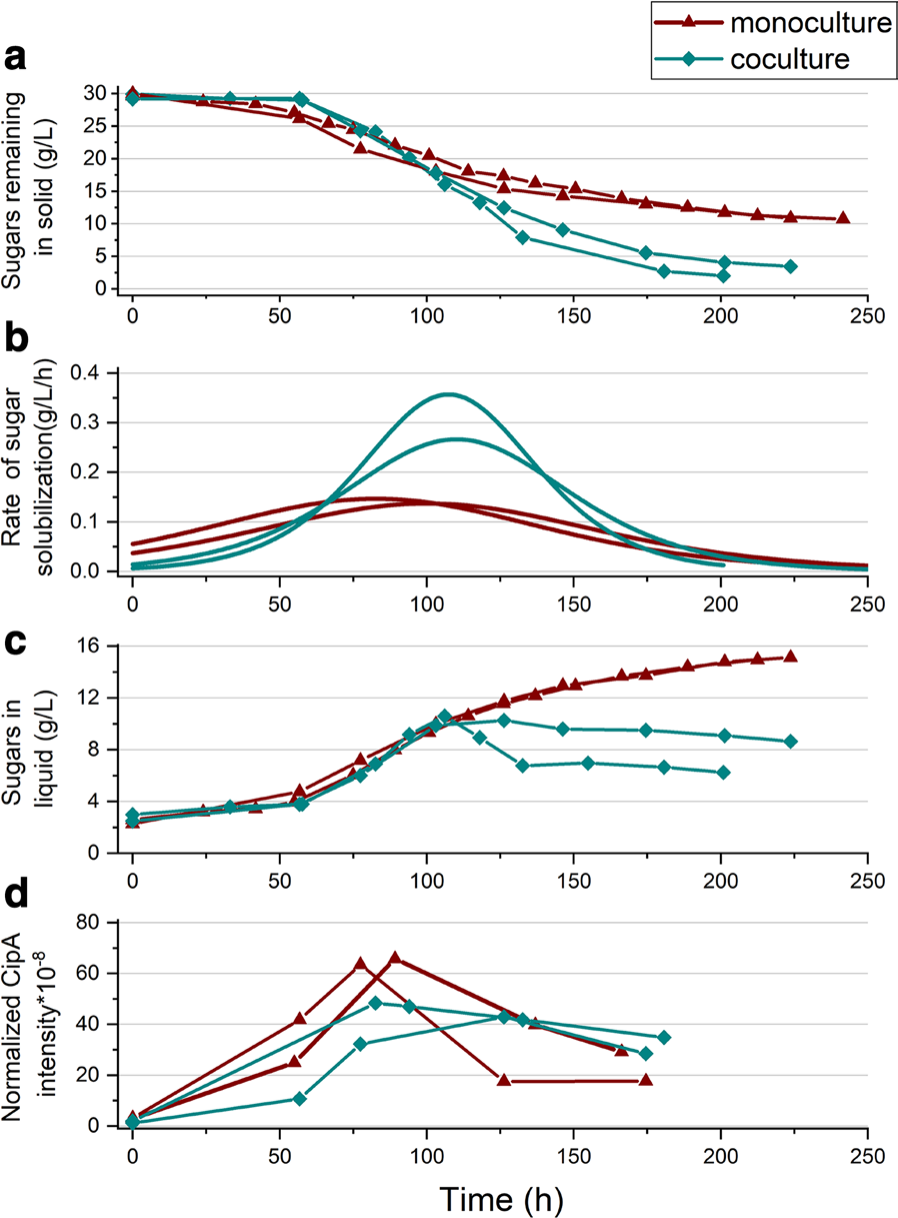
Comparison of monoculture and coculture in fermenting corn fiber. Fermentation data for *C. thermocellum* monoculture and its coculture with LL1355 and LL1548 on 40 g/L corn fiber in bioreactors: **a** Sugars remaining in solid (g/L); **b** rate of solubilization (g/L/h); **c** sugars in liquid (g/L); **d** biocatalyst as determined by normalized CipA intensity. Cocultures were inoculated with LL1355 and LL1548 at 0 and again at 50 h

Figure 7a shows that the cocultures performed much better than the monocultures at 40 g/L solids, with solubilization of 88–93% and 67%, respectively. One fermentation solubilized 93% of the corn fiber in 200 h despite an initial lag of 50 h. The maximum solubilization rate (Fig. 7b) was more than two times higher in the coculture compared to the monoculture. More rapid and extensive solubilization was associated with (and may be directly related to) the extent of hemicellulose utilization. In the better performing of the duplicate coculture fermentations, 68% of the total solubilized hemicellulose hydrolysis products (amount of sugars measured by liquid QS) were utilized, while in the other fermentation, this value was only 54%. Figure 7c shows much higher accumulation of soluble carbohydrate in the monoculture. This soluble carbohydrate was measured by liquid QS and was ~ 90% oligomeric based on the measurement of monosaccharides by HPLC. Also, the sugar composition was > 85% arabinose + xylose + galactose. The small amount of glucose is likely from the xyloglucan in corn fiber. The solubilization rate declined in the monoculture once the concentration of soluble carbohydrate exceeded 10 g/L. The better performance of the cocultures is in line with the results of a recent study using *C. thermocellum* by Froese et al. [34]. They showed that cocultures of *C. thermocellum* with *Clostridium stercorarium* and *Thermoanaerobacter thermohydrosulfuricus* were able to solubilize 58% more of the total polysaccharides in wheat straw compared to the *C. thermocellum* monoculture control.

We expected that the coculture organisms would allow further growth of *C. thermocellum* by consuming the potential inhibitors, but this was not the case (Fig. 7d). In fact, the maximum CipA intensity, taken as an indication of *C. thermocellum* concentration, was slightly higher for the monoculture. The coculture had less *C. thermocellum*, yet solubilized more of the corn fiber. However, it is noteworthy that the decrease in CipA seen in the monoculture after the maximum level at 90–100 h is less apparent in the coculture, in which CipA levels remain relatively stable. In line with this observation, the spent broths resulting from these fermentations were tested for inhibition of *C. thermocellum* growth on cellobiose and the coculture broth was distinctly less inhibitory than the monoculture broth (Additional file 1: Figure S6).

The improved performance of the coculture is not simply due to lower levels of total hemicellulose sugars. As can be seen in Fig. 7 at about 100 h when both reactors are around their peak rate of solubilization, the level of accumulated hemicellulose sugars is approximately the same. However, the rate of solubilization for the coculture at this stage is more than two times that of the monoculture. Corn fiber hemicellulose released by *C. thermocellum* is structurally heterogeneous [26], and the sum of total sugars we measured may not reveal the level of the particular molecular species that are inhibitory. In addition, there may be inhibitors other than hemicellulose that accumulate during corn fiber solubilization that are removed by the coculture partners.

In experiments to explore what the inhibitors in the spent broth could be, we observed that the filtrate through a 10 kDa molecular weight cut-off filter, which contained ~ 85% of the carbohydrates, was not inhibitory to growth on cellobiose, but the retentate was. This indicates that the inhibitor is of high molecular weight and raises the possibility that it may not be a carbohydrate. Our inability to replicate cellobiose growth inhibition using any of the hemicellulose oligosaccharides we tested supports this possibility. In further experiments, a hydrolysate prepared by incubating *C. thermocellum* cellulase with corn fiber was also strongly inhibitory to growth on cellobiose, indicating that the inhibitor was released from corn fiber and not produced from fermentation.

A component of corn fiber that we did not examine are phenolic compounds such as ferulic or coumaric acids. These are known to inhibit cellulases and reduce enzymatic hydrolysis of corn pericarp [35]. They were also suggested as potential inhibitors in the work of Basen et al. on *C. bescii* [14]. *C. thermocellum* has been shown to liberate coumaric acid from lignocellulosic biomass [36], so it is possible that their accumulation during corn fiber fermentation could be inhibiting *C. thermocellum*. These compounds could also have interacted with the PES membrane of the MWCO filter, held in the retentate, and complicated their analysis.

## Conclusions

We show here that there is a sharp decline in *C. thermocellum* solubilization of corn fiber at loadings of 40 g/L, declining from 95% at 10 g/L to 67% at 40 g/L (in 4 and 10 days, respectively). Also, the spent broth from the 40 g/L corn fiber fermentation is inhibitory to the cellulase activity of *C. thermocellum*. This is partly explained by the buildup of hemicellulose hydrolysis products, especially arabinoxylan and XOS, in the liquid broth. However, the spent broth also inhibited growth on cellobiose, which could not be replicated with any of the model carbohydrates we tested. Therefore, there is an undiscovered inhibitory factor produced in corn fiber fermentations that requires further characterization. Xylooligomers are well-known inhibitors of cellulase enzymes which may be explained by the structural similarity between xylobiose and cellobiose. In microbial communities in nature, accumulation of hemicellulose would be unexpected, since hemicellulose-consuming organisms are typically present. It is plausible that in natural environments, there is no evolutionary pressure for C6-fermenting organisms like *C. thermocellum* to overcome inhibition by C5 sugars.

The addition of coculture partners restored fermentation performance of *C. thermocellum*, but the exact mechanism remains unclear. The removal of solubilized hemicellulose carbohydrates is likely to be part of the story, but the particular molecules involved are not yet defined. Enzymatic treatment of spent broths may detoxify and shed light on this topic, especially using enzymes prepared from *Herbinix* spp. strain LL1355. The possibility of phenolic compounds being a major inhibitor will be a worthwhile investigation. Nonetheless, inhibition by solubilized hemicellulose is likely to emerge as a significant barrier to fermentations of lignocellulosic feedstocks at high solids loading. Developing suitable coculture organisms to partner with *C. thermocellum* may be an important line of future research. In some cases, specialized organisms may need to be isolated, as *Herbinix* spp. strain LL1355 was isolated for consumption of corn fiber hemicellulose, but they will need to be engineered for high ethanol yield or their enzymatic capabilities transferred to ethanologens.

## Materials and methods

### Strains, media, and fermentation conditions

Fermentations were carried out with *C. thermocellum* strain LL1043, also known as M1570 [37]; the M1442 strain of *T. saccharolyticum* [38] and various derivatives of *T. thermosaccharolyticum* strain LL1244, also known as ATCC 31960 and HG-8. LL1355 was a novel isolate as reported elsewhere [26].

Destarched corn bran and growth media were prepared as previously described [26]. The fermentations were carried out as described previously [39] in 3 L (2-L working volume) Biostat A-plus bioreactors (Sartorius Stedim, Bohemia, NY) with the temperature maintained at 55 °C using a resistive heat blanket and stirred at 200 rpm. The pH was controlled at 6.95 with a Mettler-Toledo pH probe (Columbus, OH) by the addition of 4N KOH. Base addition, pH, and temperature were recorded as described previously [40]. The reactors were autoclaved for 1 h in a liquid cycle with only the feedstock and water in the bioreactor. Biomass–water slurries were sparged with a mixture of 95% nitrogen and 5% carbon dioxide (Airgas Northeast, White River Junction, VT) while cooling to 55 °C. Medium components were then added into the reactor and pH was adjusted to 6.95 before inoculating. The inocula were from a *C. thermocellum* bioreactor fermentation on 20 g/L Avicel, harvested at the time of its peak gas production rate, and frozen in 50 mL aliquots in N2-flushed 150 mL serum bottles at − 80 °C. A 1% inoculum was used for all reactors other than one of the 10 g/L reactors which used a 5% inoculum. Samples were taken after first clearing the lines by drawing and discarding 3 mL of culture, then withdrawing a ~ 60 mL sample. While this sample was being stirred, three samples of 1 mL were taken for CipA measurements. 50 mL of sample was then measured into a Falcon centrifuge tube (Corning Inc., Corning, NY). The tubes were centrifuged for 15 min at 7000×*g* to separate solids and the liquid was pipetted out. This liquid was then used for liquid Quantitative Saccharification (QS) as described below. The solids were washed twice with DI water before being dried in a 60 °C oven till constant weight, which was noted. The residual sugars in these solids were measured by solids QS in triplicate, as described below.

For the coculture reactors, *T. thermosaccharolyticum* was grown in CTFUD media [41], pH 6.7 with 5 g/L cellobiose. LL1355 inoculum was prepared in CTFUD media supplemented with 1.5 mg/L folate and 0.1 mg/L vitamin B12 (Sigma-Aldrich). A 3% inoculum of each organism was used.

### Testing inhibition by spent broth

The spent broth collected at each sampling time point of the fermentation was centrifuged to remove solids and then filtered through a 0.22 µm syringe filter (Corning Inc., Corning, NY) into a sterile tube. 10 g/L MOPS as buffer (pH adjusted to 7.2), fresh CC6 [26] media components, and 5 g/L cellobiose were added. A 2% inoculum of *C. thermocellum* grown on CC6 media was used. A control broth was prepared from a *C. thermocellum* fermentation on 4.2 g/L Avicel.

### Carbohydrates

The source of each is given in Additional file 1: Table S2. Carbohydrate solutions were made separately, filter-sterilized into serum bottles, and purged with 20 × 45 s of alternating cycles of vacuum and nitrogen to remove oxygen. Xylooligosaccharides (Xylan from Corn Core) were obtained from TCI Chemicals (product # X0078). Wheat arabinoxylan, pullulan, and arabinan had to be autoclaved as their solutions were too viscous to filter. Glucomannan and galactomannan were hydrolyzed to oligomers by incubating a 2% (w/w) slurry with 20 Units/g (substrate) loading of β-mannanase (Megazyme, product # E-BMACJ) for 24 h at 37 °C followed by precipitating the polymers with 60% ethanol. Xyloglucan was similarly hydrolyzed using a xyloglucanase (Megazyme, product # E-XEGP), but precipitation was done using 70% ethanol instead.

### Analytical methods

The dry residual solids from a fermentation were analyzed using complete acid hydrolysis and HPLC (Quantitative Saccharification—QS) as described [40]. The total soluble sugars in the liquid broth were analyzed by a similar acid hydrolysis method we refer to as liquid QS: broth was centrifuged to remove solids and 35 µL of 72% H_2_SO_4_ was added to 1 mL of clear liquid supernatant, then put in a 4 mL shorty vial (DWK Life Sciences, Millville, NJ), and capped with a butyl rubber stopper. These vials were then autoclaved for 1 h on a liquid cycle, along with acidified sugar standards to control for degradation. All polysaccharides/oligosaccharides in the liquid were thus hydrolyzed and then measured on HPLC (Waters, Milford, MA) using xylose, arabinose, and glucose HPLC standards. The separation was performed with an Aminex HPX-87H column (Bio-Rad, Hercules, CA) at 60 °C, with RI (refractive index) detection and a 5 mM sulfuric acid solution eluent at a flow rate of 0.6 mL/min. The fermentation products were measured using the same column, as described previously [27].

### CipA measurements using LC–MS/MS

The 1 mL sample taken from the reactor was washed twice to completely remove the supernatant and only retain intact cells in the pellet. Cells were lysed via bead beating in SDS lysis buffer (4% SDS, 100 mM Tris pH 8.0) followed by denaturation (10 min at 95 °C), reduction, and alkylation with DTT (10 mM) and IAA (30 mM), respectively. Proteins were extracted using chloroform:methanol as previously described [42], dried, and resuspended in 4% sodium deoxycholate, 100 mM ammonium bicarbonate buffer. Equal volumes (250 µL) of protein were digested with 2 µg of trypsin and SDS removed using ethyl acetate as previously described [43]. Tryptic peptides were resuspended in LC–MS-grade H_2_O, 0.1% formic acid spiked with heavy-labeled CipA peptide standards (~ 2.5 pmol/µL of 3 peptide standards: VEIPITLK, SFDTAIYPDR, and NDWSNYTQSNDYSFK). Five microliters of each sample were autosampled onto an in-house pulled nanospray emitter (75 µm ID) packed with 30 cm C18 resin (Kinetex 5 µm; Phenomenex) and analyzed by 1D LC–MS/MS as previously described [44].

The *C. thermocellum* proteome sequence was concatenated with common contaminant proteins (e.g., trypsin and human keratin) and reversed sequences of the target database. MS/MS data were searched against these sequences using Tide search [45] using the following parameters: parent mass tolerance of 10 ppm, a static modification on cysteine (+ 57.0214 Da), and a dynamic modification to an oxidation (+ 15.9949 Da) of methionine and (+ 8.014199 Da) on Lysine and (+ 10.008269 Da) on Arginine to address the labeled peptides (rest parameters were assigned as default), followed by filtering using Percolator [46]. Retention times of each PSM were extracted by parsing mzML files with an in-house script and MS1 apex intensities were assigned using moFF [47]. The moFF parameters were set to 10 ppm for the precursor mass tolerance, 3 min for the XIC time window, and 0.75 min (equivalent to 45 s) to get the apex for the ms2 peptide/feature.

CipA intensity was calculated by summing all CipA peptide intensities, excluding the labeled peptides which were used for sample normalization. Similarly, whole-cell proteome intensity was also calculated for each sample.

### Growth experiments on cellobiose

Growth rate measurements were performed in a Powerwave XS plate reader (BioTek Corporation, Winooski, VT) in an anaerobic chamber at 55 °C as previously described [48]. The media used for growth tests was CC6 [26] with 5–10 g/L cellobiose and 10 g/L MOPS. A 2% inoculum grown on the same medium was used.

### Growth experiments on Avicel

The Avicel growth experiments were run in 125 mL serum bottles with a final culture volume of 30 or 50 mL. The bottles with water and Avicel were sealed and purged with 20 × 45 s of alternating cycles of vacuum and nitrogen before autoclaving for 20 min on a liquid cycle. CC6 medium was used with a MOPS concentration of 10 g/L with the final pH of 6.9–7.0. The residual Avicel and pellet nitrogen was measured using the total carbon and nitrogen (TCN) analyzer, Shimadzu Scientific Instruments (Columbia, MD) as described earlier [28].

### Cellulase enzyme assays

The cellulase enzyme was prepared from a *C. thermocellum* culture grown on 20 g/L Avicel for 5 days until the substrate was completely consumed. The liquid broth was centrifuged to remove cells and filtered in an anaerobic glove bag (Coy Laboratory Products, Grass Lake, MI) to limit the exposure to oxygen. The filtered broth was then passed through a 50 kDa Molecular Weight Cut-off Filter (Millipore Sigma) to concentrate the enzymes four-to-fivefold. The protein concentration was measured using a standard Bradford assay as described earlier [26]. The 5 mL assay consisted of 0.25 mL 1 M citrate buffer (pH 5.5), 0.25 mL of 0.1 M DTT (dithiothreitol) solution, 0.25 mL of 0.2 M CaCl_2_ solution, various enzyme concentrations, and water.

The qualitative assays were run with 0.5–1 g/L initial Avicel. To estimate the remaining Avicel, the absorbance was measured using a portable spectrophotometer (Biochrom Colorwave WPA CO7500) at a wavelength of 600 nm. The quantitative assays were run with 6 g/L initial Avicel. The enzyme loading was 10–25 mg protein/g of Avicel. Samples were taken to determine glucose released from Avicel by performing liquid QS as described above. Liquid QS allowed for the hydrolysis of all the released glucose oligomers into monomeric glucose that was then measured by HPLC.

## Supplementary Information

**Additional file 1: Figure S1.** Timecourse of glucose release from Avicel for cellulase assay with the addition of spent broth. **Figure S2.** Total sugars in fermentation broth from *C. thermocellum* monoculture reactors on corn fiber, measured by liquid QS. **Table S1.** List of carbohydrates tested for effect on growth rate of *C. thermocellum* on cellobiose. **Table S2.** Sources of carbohydrates used in the study. **Figure S3.** Effect of different monosaccharides on *C. thermocellum* cellulase activity. **Figure S4.** Effect of adding Xylooligosaccharides to *C. thermocellum* Avicel fermentations in bioreactors. **Figure S5.** Curve fitting to measure the effect of various carbohydrates on the utilization of Avicel by *C. thermocellum*. **Figure S6.** Effect of final spent broth from a corn fiber coculture fermentation on growth of *C. thermocellum* on cellobiose in a plate reader.

**Additional file 2.** Supporting data for Fig. 6. Avicel remaining over time is given for Avicel fermentations with addition of various carbohydrates, along with curve fits used to calculate maximum Avicel utilization rate. 

## Data Availability

All data generated or analyzed during this study are included in this published article and its Additional files.
