# Supplementary material for: Coculture with hemicellulose-fermenting microbes reverses inhibition of corn fiber solubilization by Clostridium thermocellum at elevated solids loadings
Source: Biotechnol Biofuels. 2021 Jan 18;14:24. doi: 10.1186/s13068-020-01867-w (PMC7814735; doi:10.1186/s13068-020-01867-w)
Supplement: Supplementary file 1 — Additional file 1: Figure S1. Timecourse of glucose release from Avicel for cellulase assay with the addition of spent broth. Figure S2. Total sugars in fermentation broth from C. thermocellum monoculture reactors on corn fiber, measured by liquid QS. Table S1. List of carbohydrates tested for effect on growth rate of C. thermocellum on cellobiose. Table S2. Sources of carbohydrates used in the study. Figure S3. Effect of different monosaccharides on C. thermocellum cellulase activity. Figure S4. Effect of adding Xylooligosaccharides to C. thermocellum Avicel fermentations in bioreactors. Figure S5. Curve fitting to measure the effect of various carbohydrates on the utilization of Avicel by C. thermocellum. Figure S6. Effect of final spent broth from a corn fiber coculture fermentation on growth of C. thermocellum on cellobiose in a plate reader. [file 13068_2020_1867_MOESM1_ESM.pdf]

Additional File 1, Supplementary Figures and Tables

Beri et al., Coculture with hemicellulose-fermenting microbes reverses inhibition of corn fiber solubilization by *Clostridium thermocellum* at elevated solids loadings

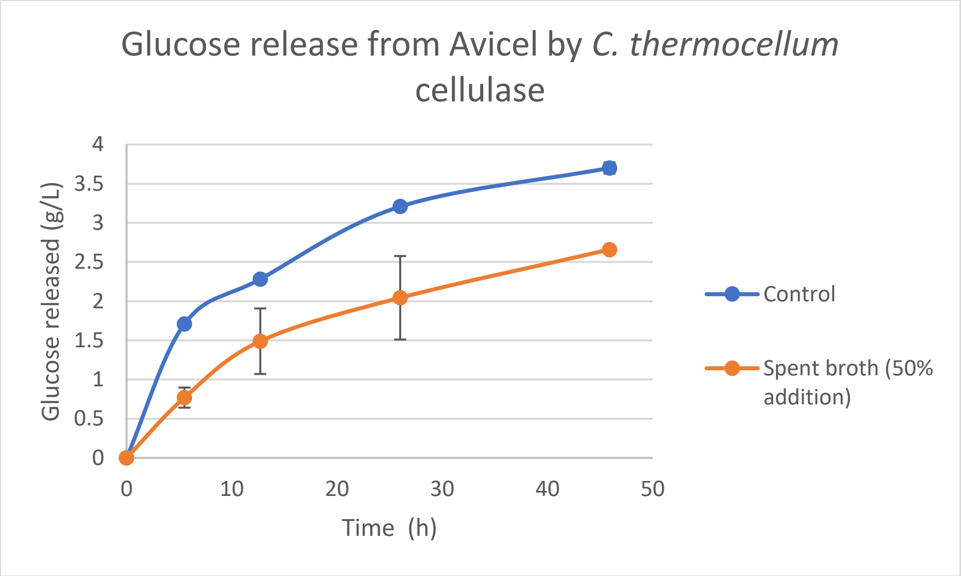

Figure S1- Timecourse of glucose release from Avicel for cellulase assay with the addition of spent broth- 20 mg (cellulase)/g (Avicel) was incubated with 6 g/L Avicel for 50 h. Regular samples were taken to measure the release of glucose by liquid QS.

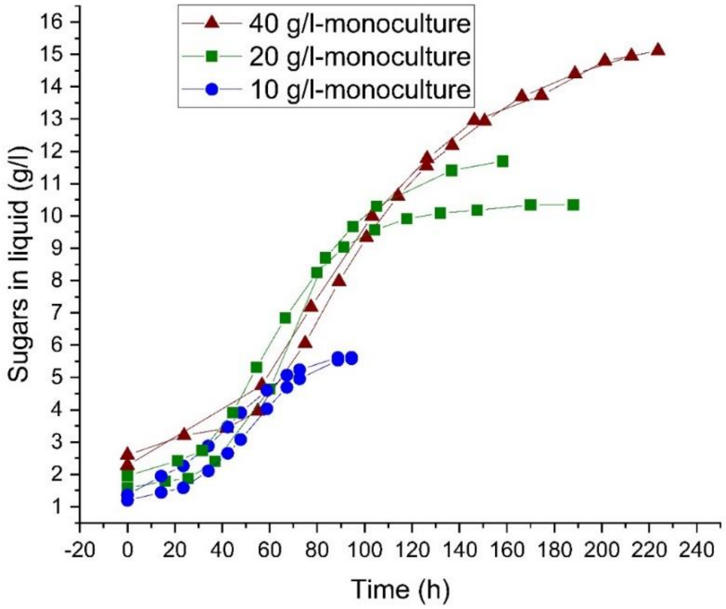

Figure S2- Total sugars in fermentation broth from *C. thermocellum* monoculture reactors on corn fiber, measured by liquid QS.

Monosaccharides	Disaccharides	Oligosaccharides	Polysaccharides
D-Xylose	Sucrose	XOS	Pullulan
Arabinose	Xylobiose	Xyloglucan	Inulin
Glucose	Mannobiose	Glucomannan	Arabinan
Fructose	Maltose	Galactomannan	Wheat AX
D-Galactose	Melibiose	Stachyose	
L-xylose		A^3X	
L-Rhamnose		A^2XX	
D-ribose		Xylotriose	
L-sorbose		Xylotetraose	

Table S1- List of carbohydrates tested for effect on growth rate of *C. thermocellum* on cellobiose. XOS- Xylooligosaccharides, AX- arabinoxylan, A^3X- 3^2-α-L-Arabinofuranosyl-xylobiose, A2XX- 2^3-α-L-Arabinofuranosyl-xylotriose

Sigma-Aldrich	D-Xylose, L-Xylose, Glucose, Fructose, D-galactose, Sucrose, Xylan from Birchwood
Megazyme	Mannobiose, Glucomannan (Konjac;Low Vis), A^3X- 3^2-α-L-Arabinofuranosyl-xylobiose, A2XX- 2^3-α-L-Arabinofuranosyl-xylotriose, Wheat Arabinoxylan (Wheat Flour; Insoluble), Xylotriose, Xylotetraose, Arabinan (Sugar Beet), Xylan from Beechwood
Carbosynth	L-Rhamnose monohydrate, L-Sorbose, Xylobiose, D-Maltose monohydrate, D-Melibiose monohydrate, Inulin, Xyloglucan, Pullulan, D-Galacto-D-mannan- from carob, Stachyose hydrate
TCI America	Xylan from corn core

Table S2- Sources of carbohydrates used in the study

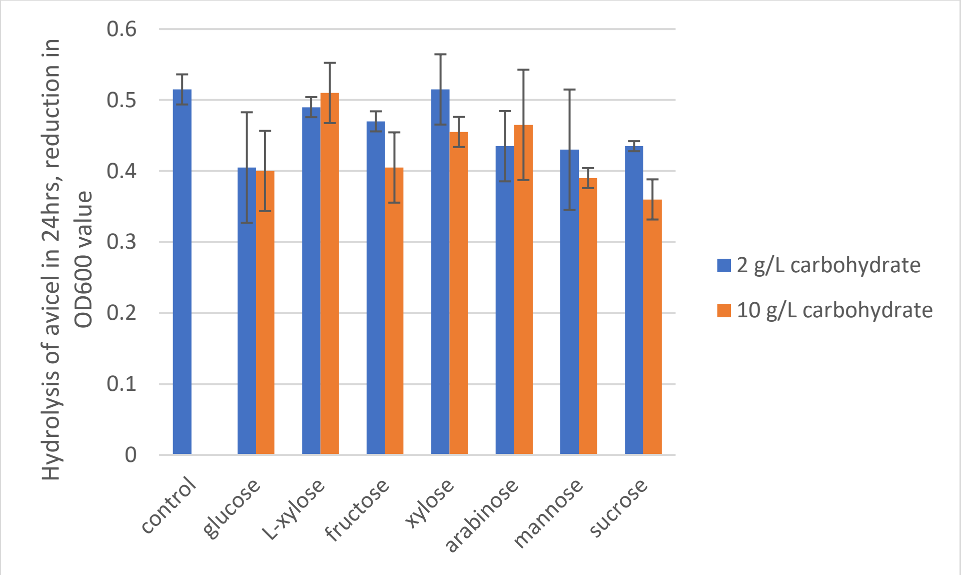

Figure S3- Effect of different monosaccharides on *C. thermocellum* cellulase activity - 1 g/L initial Avicel was incubated with 10 mg/g *C. thermocellum* cellulase enzyme and activity was estimated by measuring the decrease in OD_{600} after 24h. Carbohydrates were added at two different concentrations of 2 g/L and 10 g/L. The error bars show the SD for n=2.

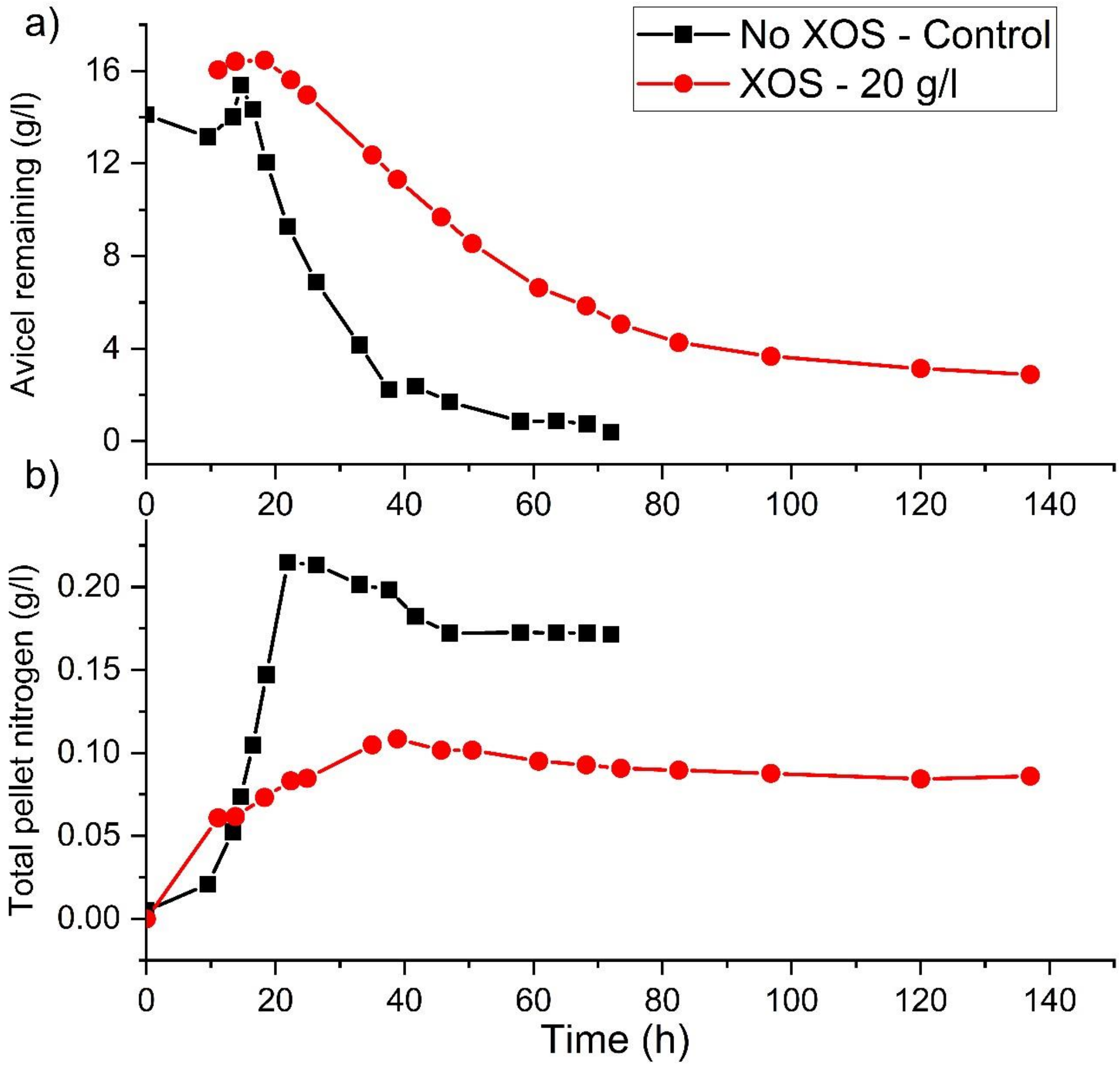

Figure S4- Effect of adding Xylooligosaccharides to *C. thermocellum* Avicel fermentations in bioreactors. Fermentation data from two pH controlled fermentors showing a) Avicel remaining and b) total pellet nitrogen for *C. thermocellum* growth on 20 g/l Avicel with and without the addition of 20 g/l XOS.

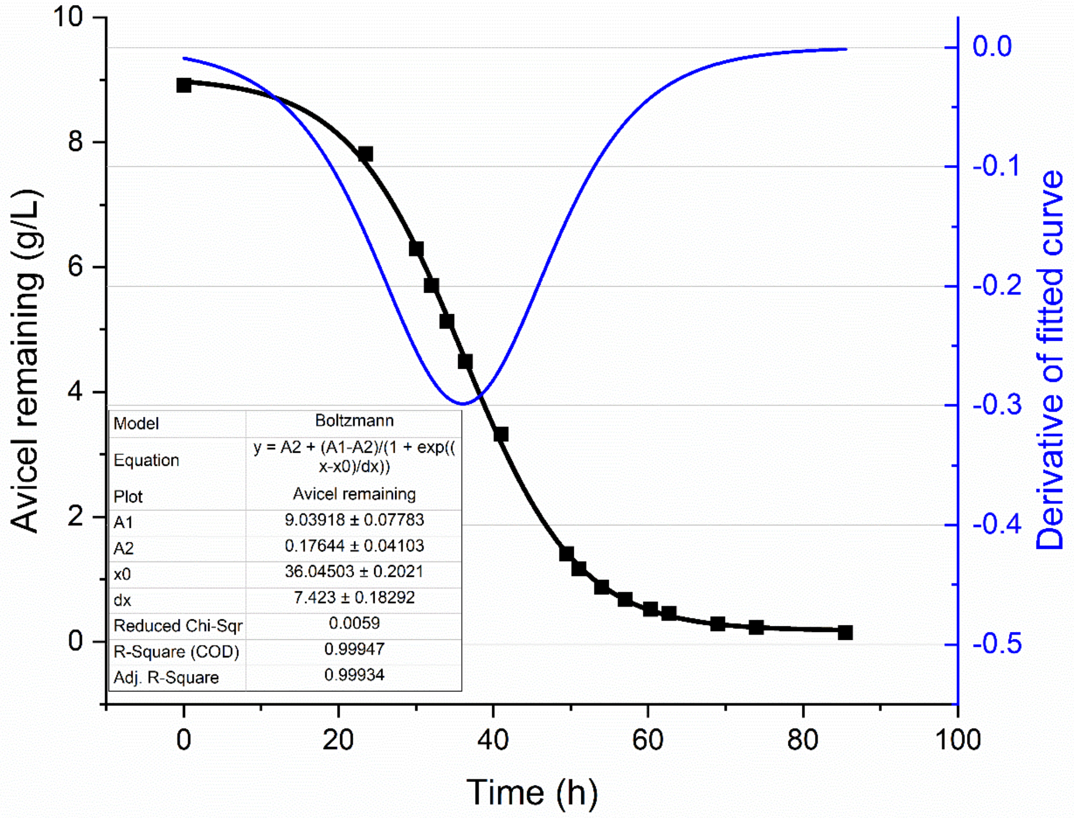

Figure S5- Curve fitting to measure the effect of various carbohydrates on the utilization of Avicel by *C. thermocellum*. Substrate utilization by *C. thermocellum* at 10 g/l Avicel loading was measured using a total carbon and nitrogen (TCN) analyzer. Curves were generated with Boltzmann Sigmoidal fitting and the derivative taken to calculate maximum rate of Avicel utilization. Figure 6 in the main text shows calculated rates with the addition of various carbohydrates at a 10 g/l concentration. The data for one of the control replicates is shown here, and the rest of the data are provided in Additional file 2.

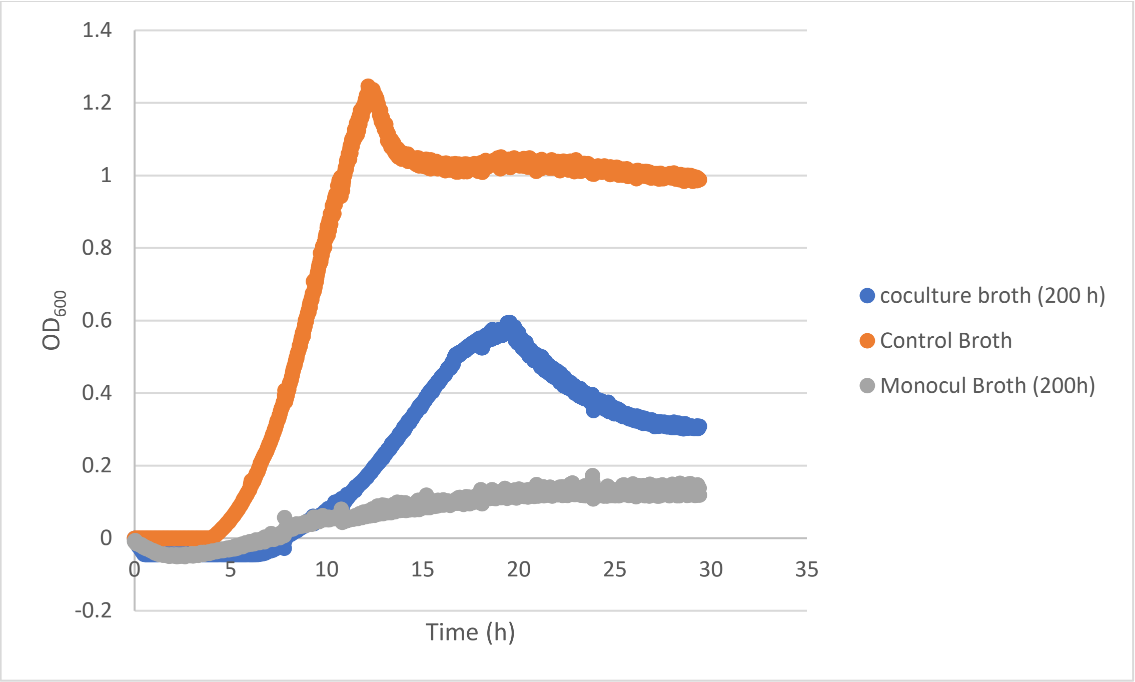

Figure S6- Effect of final spent broth from a corn fiber coculture fermentation on growth of *C. thermocellum* on cellobiose in a plate reader.
